# Confidence from uncertainty - A multi-target drug screening method from robust control theory

**DOI:** 10.1186/1752-0509-4-161

**Published:** 2010-11-24

**Authors:** Camilla Luni, Jason E Shoemaker, Kevin R Sanft, Linda R Petzold, Francis J Doyle

**Affiliations:** 1 Department of Chemical Engineering, University of California, Santa Barbara, CA 93106-5080, USA; 2 Department of Computer Science, University of California, Santa Barbara, CA 93106-5070, USA

## Abstract

**Background:**

Robustness is a recognized feature of biological systems that evolved as a defence to environmental variability. Complex diseases such as diabetes, cancer, bacterial and viral infections, exploit the same mechanisms that allow for robust behaviour in healthy conditions to ensure their own continuance. Single drug therapies, while generally potent regulators of their specific protein/gene targets, often fail to counter the robustness of the disease in question. Multi-drug therapies offer a powerful means to restore disrupted biological networks, by targeting the subsystem of interest while preventing the diseased network from reconciling through available, redundant mechanisms. Modelling techniques are needed to manage the high number of combinatorial possibilities arising in multi-drug therapeutic design, and identify synergistic targets that are robust to system uncertainty.

**Results:**

We present the application of a method from robust control theory, Structured Singular Value or μ- analysis, to identify highly effective multi-drug therapies by using robustness in the face of uncertainty as a new means of target discrimination. We illustrate the method by means of a case study of a negative feedback network motif subject to parametric uncertainty.

**Conclusions:**

The paper contributes to the development of effective methods for drug screening in the context of network modelling affected by parametric uncertainty. The results have wide applicability for the analysis of different sources of uncertainty like noise experienced in the data, neglected dynamics, or intrinsic biological variability.

## Background

Biological systems are hierarchically organized, from genes to proteins up to the organism level. At the cellular level, complex interconnected networks include metabolic signalling, signal transduction, and transcriptional regulatory networks [1]. Some general features of biological networks have been explored computationally, such as robustness [2], modularity [3], control coefficients [4], and connectivity properties [5]. Robustness is defined as the ability to maintain functional performance in the presence of uncertainty [2,6], and it is particularly relevant in therapy design as drug effectiveness should be independent from predictable sources of variability.

Complex diseases often exploit the same strategies present in healthy networks to gain a robust status [2]. Diseases such as diabetes, cancer, bacterial and viral infections, represent multiple disruptions within the host network structure rather than single events, such as a DNA point mutation [7]. Signalling redundancy, feedback, and other network strategies adopted by the disease, ensure that it will be robust to disturbances within its architecture. Hence, single-target therapies fail in many cases because network characteristics are not accounted for during target identification [8,9]. On the other hand, multi-drug therapies (MDT) have been proven to be effective for many complex diseases [10,11]. Network-based design of MDTs can improve current drug regimes [11-14] by identifying targets that both moderate the immediate characteristics of the disease while disarming its robustness strategies [7]. Furthermore, synergy within MDTs may reduce the required drug load, hopefully minimizing side effects [15,16].

Some MDTs are currently used to treat chronic diseases and to boost antibiotic potency. AIDS infections routinely require a drug regimen of reverse-transcriptase inhibitors and protease inhibitors [17]. Oncological chemotherapeutic regimens often involve the combination of cyclophosphamide, hydroxydaunorubicin, oncovin, and prednisone, abbreviated as CHOP [18]. Augmentin, an amoxicillin-based antibiotic, contains clavulanic acid to inhibit a known mechanism of amoxicillin degradation [19]. In comparison to their single-perturbation counterparts, these MDTs often show an order of magnitude greater efficacy [17]. Most MDTs to date have been identified in an *ad hoc *fashion, relying on observational studies of previously available drug lines. Many pharmaceutical companies are now embracing the idea of *a priori *design of MDTs using *in silico *modelling and analysis to rapidly identify candidate targets [20].

Optimizing drug combinations and concentrations produces an unmanageable number of possible therapies to explore, demanding efficient computational methods of screening [11]. Furthermore, it is unreliable to extrapolate the therapeutic efficacy from the necessarily few conditions tested. For example, a potential concentration-dependent synergistic behaviour may occur at intermediate concentrations not considered during experimentation. This situation is not unlikely considering that strongly nonlinear behaviours have been recognized in biological systems, such as switching or bistabilities [21].

For drug screening to succeed, additional insight into the biological mechanisms of drug action at the cellular level is needed to increase the predictability of the therapy. High-throughput experimental techniques are providing the data required to understand the connections between the biochemical nodes in the cellular sub-networks underlying specific functions. The causal relationships between these components are being explored by dynamic modelling through a continuous process of expansion and refinement. The most widely-used representation of the biochemical reaction network is a dynamic and continuous description, based on a system of ordinary differential equations (ODEs) [22], where the variables represent the concentrations of the components, and their change over time is simulated. Many ODE models are currently under development to gain insight into complex diseases, such as diabetes [23-25], and will be invaluable for future drug discovery, as reviewed elsewhere [26-28]. More than 200 network models from the literature have been curated and included in publicly accessible databases, such as Biomodels, BioPax and the CellML Model Repository. Systems Biology Markup Language (SBML) was created to standardize the description of biochemical networks, enabling communication between people and software [29], and paving the way for a biochemically detailed artificial organism reconstruction [30]. ODE models can be interrogated to test hypotheses of cellular response to drug combinations, considering whole sets dosage permutations and used to discover optimal points of manipulation within the network [13,16]. These models have the potential for *in silico *testing MDTs at reduced cost and time [11]. Despite improvements in the accuracy of biological models, their reliability is often limited by parameter uncertainty. Even at best, parameter values can be inferred by experimental data as a range of values, rather than a fixed one. While increasingly precise experimental measurement methods are being developed, cell-cell heterogeneity in tissues and stochastic noise, the consequence of the small copy number of some intracellular components, are intrinsic sources of uncertainty and require *ad hoc *methods of analysis.

**Figure 1:**
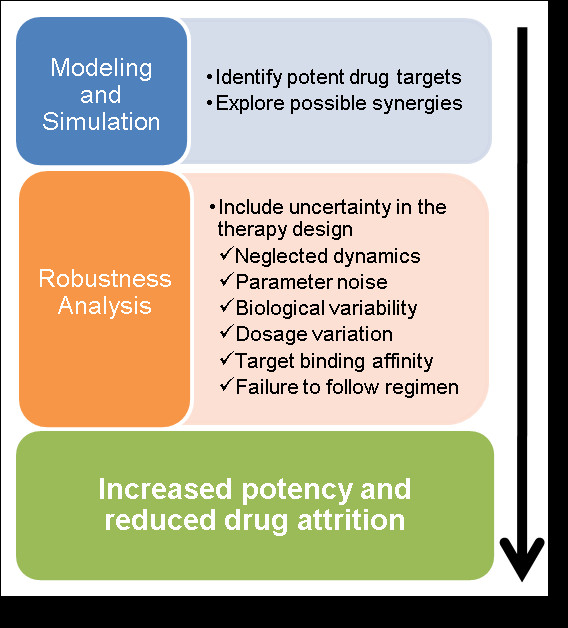
**Range of applicability of SSV analysis for robust therapy design**. Once a model has been established that satisfactorily explains the dynamics of the diseased state, SSV analysis can be used to identify potent and robust multi-drug therapy candidates. SSV analysis first identifies which therapies can best manipulate the protein(s) of interest. Then, the candidate list is further filtered to therapies which are robust to known or perceived uncertainty affecting the treatment. The uncertainty may include parameter uncertainty and uncertainty generated during model development, but also disturbances occurring during the actual treatment, such as failure to properly adhere to a drug regimen schedule.

We propose the use of Structured Singular Value (SSV) analysis as a powerful tool for drug target discrimination in biological models also accounting for uncertainty. This technique was developed in the control theory field [31], but has already been successively applied in the analysis of biological systems [32-35]. In the proposed methodology, SSV allows the discrimination of highly effective MDTs from a large pool of potential candidates, according to the robust response of the diseased network in the face of known or inferred sources of uncertainty (Figure 1). For illustration purposes, we explain the methodology through a case study, given by a negative feedback network motif. Moreover, we discuss strengths, limitations, and extensions (Figure 1) of the proposed method and its application, with respect to other existing ones.

## Results

### Case study description

**Figure 2:**
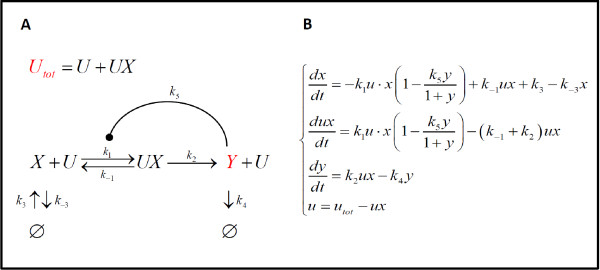
**Case study model**. (**A**) Topology of the network. *U *is the enzyme catalyzing the conversion of *X *to product *Y*, through the intermediate *UX*. The open arrows indicate the chemical reactions, and the oval arrow a negative regulation. k's represent the parameters involved in each step. *Ø *is the null component to indicate production and degradation. Input to the system is given by the total enzyme, *U_tot_*, concentration, constant over time and given by *u_tot _= u+ux *(lower-case component names indicate the corresponding concentrations). Output of the system is *y*. Inputs and outputs are highlighted in red. (**B**) Nonlinear model equations. The reaction rates are given by mass-action, negative feedback is described by the multiplicative term containing *k_5_*. The nominal values of the parameters are: *k_1 _*= 1, *k_2 _*= 2, *k_3 _*= 10, *k_4 _*= 0.5, *k_5 _*= 0.5, *k_-1 _*= 3, *k_-3 _*= 1.

A schematic description of the case study used to illustrate the proposed methodology is shown in Figure 2A. The component *X *is converted to *Y *through an enzymatic reaction, catalyzed by *U*, that includes the intermediate production of the complex *UX*. The production of *X *and the degradation of *X *and *Y *are also considered. All reaction rates are modelled by mass action. The product *Y *regulates its own production via autoinhibition. This negative feedback mechanism is modelled as a multiplicative factor dependent on the concentration of *Y*. Negative feedback is a widespread strategy in biological networks that strongly contributes to their spatial and temporal complexity [36]. The equation system is shown in Figure 2B, and the arbitrary set of nominal parameter values are provided in the figure caption.

**Figure 3:**
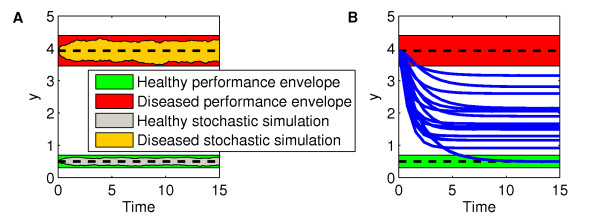
**Performance envelopes and therapeutic fitting**. (**A**) Temporal simulation of the nonlinear model in Figure 2B with nominal parameters, under healthy conditions (*u_tot,h _*= 0.2, and initial conditions given by the healthy steady-state), and diseased conditions (*u_tot,d _*= 2, and initial conditions given by the diseased steady-state). Performance envelopes are generated to contain the stochastic envelopes, resulted by the Stochastic Simulation Algorithm (mean ± standard deviation). Nominal results are also shown in dashed lines. (**B**) Comparison between the performance envelopes and the results obtained from the nonlinear model, starting from the diseased steady-state, with parameter values modified according to the 56 therapies (blue curves).

The first requirement for any drug investigation is to identify the appropriate inputs and outputs of the system. These choices depend on which components (such as cytokine concentration, mRNA level, marker expression, etc.) are significant for defining the healthy and diseased states, and are measurable by available biological assays. This example analyzes a single-input single-output system. We assume the input is the total concentration of enzyme, *u_tot_*, constant over time. A disease state emerges when the input has a high concentration, *u_tot,d_*, compared to the basal healthy state, *u_tot,h_*. As a consequence, the output concentration, *y*, is up-regulated in this condition (Figure 3A). The goal is to re-parameterize the diseased model to obtain a therapeutically treated model that, with a diseased input *u_tot,d_*, allows recovery from the diseased output to the healthy one, even in presence of uncertainty.

### Healthy performance and potential therapies

Due to biological variability, the healthy performance is given by an envelope that defines upper, *y_ub_*, and lower, *y_lb_*, bounds on the output, rather than an idealized, nominal single trajectory (Figure 3A). Thus, the system meets the requirements for healthy performance when:

(1) $$y_{lb}\leq y\leq y_{ub}.$$

In practice, the performance bounds are derived by the standard deviation of the experimental data. In this work, the system "noise" is artificially generated simulating the system with the Stochastic Simulation Algorithm, SSA [37]. A smooth performance envelope is then defined to approximately contain the concentration profiles resulting from these simulations, as shown in Figure 3A and explained in the Methods section.

Multiple therapeutic approaches can be investigated that aim at restoring the normal output concentration in the presence of a diseased input condition. A drug effect on the system can be modelled as a parameter perturbation, i.e., modifying a component's rate of synthesis, degradation, or interaction with other elements in the network. We first inferred the set of potential therapies by fitting the healthy output curve in the presence of a diseased input, *u_tot,d_*, targeting up to 4 parameters at a time. Thus, each therapy model is in the form of the equation system presented in Figure 2B, with a diseased input, *u_tot,d_*, and a different parameter set obtained solving a least-square optimization problem that minimizes the deviation of its output from the healthy one. A total of 56 possible therapies, i.e. $\sum_{i=1}^{4}\left(\begin{array}{c} n\\ i \end{array}\right)$ combinations of the *n *= 6 parameters, were obtained. A comparison between the outcome, *y_dt_*, from each therapy and the performance envelopes is shown in Figure 3B, where the simulations were performed starting from the diseased steady-state in absence of any source of uncertainty.

### Selection of therapies for nominal performance

According to the definition in (1), the criterion for nominal performance requires that the output of a therapeutic model does not cross the boundaries of the healthy performance envelope, when using the healthy steady-state as initial condition. It is formally convenient to re-formulate the problem defining an upper bound for the absolute deviation of the therapy output from the healthy one:

(2) $$\left|y_{dt}(t)-y_{h}(t)\right|\leq \frac{y_{ub}-y_{lb}}{2}.$$

**Figure 4:**
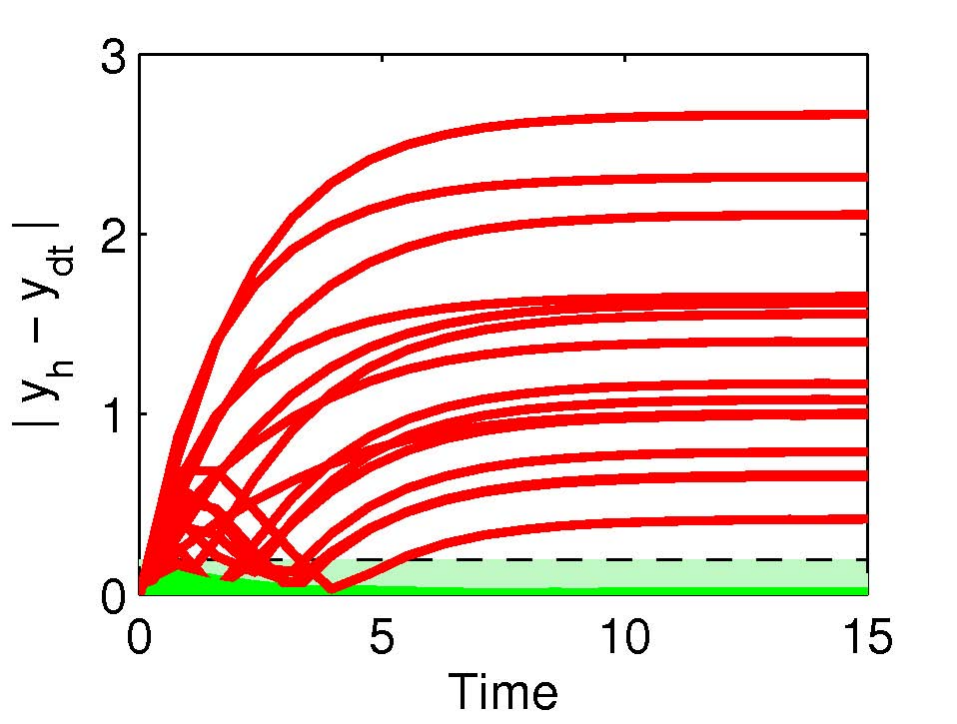
**Nominal performance analysis results**. Trajectories obtained with the nonlinear deviation model for each of the 56 therapies without parametric uncertainty. Green and red lines denote therapies that pass and do not pass the nominal performance selection criterion, respectively. The performance envelope is shaded in light green.

We applied this preliminary screening method, based on direct trajectories comparison, to our case study. A total of 41 therapies, out of 56 potential, were selected as giving a healthy nominal performance in the presence of a diseased input, *u_tot,d_*, i.e., their output without any parametric uncertainty falls within the healthy performance envelope (Figure 4).

### Uncertainty description and robust performance

A mathematical approximation of a complex biophysical system must account for multiple sources of uncertainty, due to stochastic noise, experimental error, or other possible fluctuations induced by the interaction of the system with its surrounding. A confidence interval can be assigned to each parameter, during the procedure of experimental data fitting, as a lumped measure of these multiple sources of uncertainty. Thus, each parameter in the model is represented in the following form:

(3) $$k=k_{mean}(1+r_{k}\cdot \delta_{k}),$$

where *k *∈ [*k*_min_, *k*_max_] is a generic parameter of the model, *k_mean _*= (*k*_min _+ *k*_max_)/2, *r_k _*= (k_max _- k_min_)/(k_max _+ k_min_), and *δ*_*k *_∈ ℝ and |δ_*k*_| ≤ 1. In this case study, we assume that all parameters have a relative fluctuation of 45% about their mean value (i.e., *r_k _*= 45%).

The conditions for *nominal *performance (without uncertainty) can be extended to the case of an uncertain model. Specifically, a therapy meets the criterion for *robust *performance if, for any set of parameters within the defined uncertainty range, no output trajectory crosses the healthy performance envelope boundaries, when using the healthy steady-state as initial condition. A direct comparison between each therapy's output trajectories and the healthy performance envelope, as in the previous section, is not feasible for all the values of the uncertain parameters. The advantage of employing SSV analysis becomes apparent in this situation.

### Rearrangement of the model in M-Δ form

SSV analysis is a tool developed in control theory to study the performance of systems affected by uncertainty [31]. We provide here an intuitive understanding of how it works, and refer to textbooks in the field for a more technical explanation [38]. Before SSV application, some preliminary steps are needed to recast the model in a suitable form, including model Jacobian linearization, and Laplace transforms. They are well-known techniques in control theory and numerical algorithms to perform them are readily available in technical software such as Matlab.

We defined a deviation model as the difference between a therapy model and the healthy one, and we normalized the output by a weighting factor, *w_p_*, representing the performance specification. The criterion for robust performance can now be expressed in terms of ratio between the normalized deviation model output, (*y_dt _*- *y_h_*)/w_p_, and its input,(*u_tot,d _*- *u_tot,h_*), i.e.:

(4) $$\frac{\left(y_{dt}-y_{h}\right)/w_{P}}{u_{tot,d}-u_{tot,h}}<1.$$

The performance weighting factor, *w_p_*, is related to the performance envelope bounds by the following relationship:

(5) $$w_{P}=\frac{\left(y_{ub}-y_{lb}\right)/2}{u_{tot,d}-u_{tot,h}}.$$

**Figure 5:**
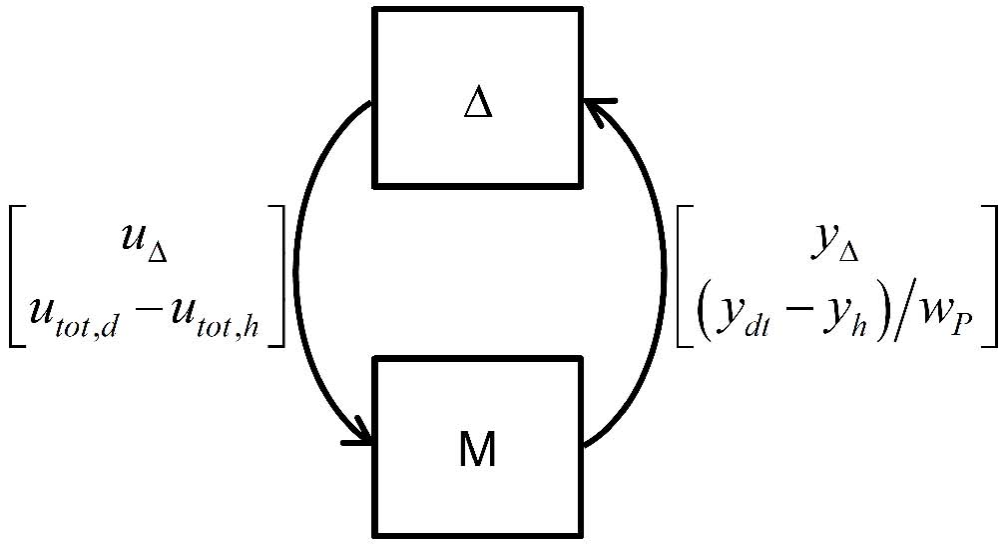
**Block diagram representation of the deviation models**. The deviation model is shown in M-Δ form. The vector of input and output between the two blocks are also indicated. *u*_Δ _and *y*_Δ _represent the uncertain components of the input and output, respectively, for the system M.

Now the model includes parametric uncertainty. It is always possible, through a linear fractional transformation (LFT), to pull out the uncertain elements from the nominal model, and to recast it in a M-Δ form (Figure 5), where M is the matrix describing the nominal model and Δ the matrix containing all the uncertainty, namely the δ_*k*_'s defined above, and the performance specification. The Δ-matrix has a particular structure, due to the presence of a number of zeros in some positions, dependent on the specific starting model. As |δ_*k*_| ≤ 1 and the output is normalized by *w_p_*, Δ is also normalized. From a control theory standpoint, putting the system in this form converts the performance analysis problem to the study of the stability of the loop in Figure 5.

### General aspects on SSV

SSV is a worst-case analysis that excludes a therapy if, even for a single parametric combination within the defined uncertainty ranges, it fails to meet the performance specifications defined by the envelope. It is based on the calculation of the structured singular value, *μ_RP _*(where RP stands for robust performance), by solving the following minimization problem:

(6) $$\mu_{RP}(M)={\left(\underset{\Delta }{\min}\{k_{m}|\det(I-k_{m}M\Delta )=0\text{for structured and normalized }\Delta )\}\right)}^{-1}$$

where I is the identity matrix, and *k_m_* a scalar factor. A result well-known in control theory, simplistically stated here, is that, when det(*I *- *MΔ*) = 0, then the loop in Figure 5 becomes unstable, i.e., in our case, the performance is not fulfilled. As Δ is a matrix whose elements are uncertain, the above minimization problem is solved over all possible Δ's that are normalized and have the structure that we mentioned in the previous section.

The value min(*k_m_*) represents the smallest perturbation that destabilizes the system, and *μ_RP _*is its reciprocal. Thus, *μ_RP _*= 1 means that there exists a perturbation, within the uncertainty description, that is large enough to pull the output exactly to the limit of the performance envelope. The model meets the conditions for *robust *performance if and only if *μ_RP _*< 1. Details on how *μ_RP _*is computed are available in the literature [38], and algorithms are also included in technical software, like Matlab.

### Selection of therapies for robust performance by SSV

**Figure 6:**
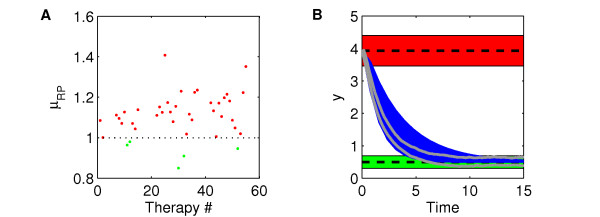
**Robust performance analysis and results of one robust therapy in presence of parametric uncertainty**. (**A**) Results of the SSV analysis applied for robust performance, in presence of parametric uncertainty. Green and red dots illustrate therapies that pass and do not pass the robust performance selection criterion, respectively. (**B**) Comparison between the performance envelopes described in Figure 3A and the results obtained from therapy no. 30. Blue curves are the simulation results by therapy 30 linearized model with 100 different parameter sets, sampled within ± 45% of the nominal values. Gray curves are the upper and lower bounds of the stochastic envelope generated by the Stochastic Simulation Algorithm of therapy 30 nonlinear model (mean ± standard deviation of 100 trajectories).

Values of *μ_RP_* for the 41 therapies are shown in Figure 6A. Only 5 therapies have *μ_RP _*< 1 and passed this screening test. Table 1 summarizes the parameters involved in each one. Interestingly, no single-parameter therapies met the robust performance specification. This is a confirmation of the importance of a MDT approach, as opposed to a drug strategy having only one target of intervention. In fact, because of the interconnected structure of the network, a robust therapy was obtained by drug (i.e. parameter) combinations affecting at the same time processes with the same effect but dislocated in different points of the network. For example, therapy 11 increases *X *concentration by reducing its consumption in the forward reaction involving parameter *k_1_*, and by reducing its degradation dependent on parameter *k_-3_*. Therapy 12 instead decreases *Y *concentration by reducing its production from *UX *(parameter *k_2_*) and increasing its degradation (parameter *k_4_*).

**Table 1:** Increase* or decrease# of parameter values in the robust therapies respect to their nominal ones

Therapy no.	11	12	30	32	52
**Affected parameters**	k_1 _↓	k_2 _↓	k_1 _↓	k_2 _↓	k_2 _↓
	
	k_-3 _↓	k_4 _↓	k_5 _↓	k_4 _↓	k_4 _↓
	
			k_-3 _↓	k_5 _↑	k_5 _↑
	
					k_-1 _↑

* Upward arrows. ^# ^Downward arrows.

Figure 6B shows the output dynamics of therapy 30. First, the nominal values of the parameters were randomly changed by up to ± 45%. 100 parameter sets were generated, and used in the linearized model to simulate the diseased treated system, with diseased steady-state as initial condition. In all cases the output steady-state value falls within the healthy performance envelope, as a confirmation of SSV analysis results. Then, the nominal nonlinear model of therapy 30 was simulated by SSA. SSV analysis does not guarantee performance in this case, as noise in component concentrations was not included in the uncertainty description, which was only parametric. Nonetheless, the stochastic envelope generated falls into the performance envelope even in this case after a transient (Figure 6B), increasing our confidence on the efficacy of this therapy in the presence of unexpected uncertainty.

## Discussion

In this paper we have proposed a new method for MDT selection, taking advantage of SSV analysis, a tool already successfully applied in other fields such as aeronautics [39]. We have evaluated the feasibility of using this tool for drug screening by a simple case study, essentially a network given by an enzymatic reaction negatively regulated by its own product. While therapies can be easily selected based on a criterion of nominal performance, the importance of SSV application is apparent in presence of parametric uncertainty, when, to the best of our knowledge, alternative methods are not available.

Through the case study, we demonstrated the relevance of considering the effect of structured uncertainty, i.e. parametric noise, as only 5 therapies, out of 41 showing nominal performance, were robust. From a network perspective, the results emphasize how MDTs offer greater potency in regulating specific targets. In fact, all the 5 therapies passing the screening involved multiple perturbations. Furthermore, these resulted in therapies that are also less susceptible to internal biological fluctuations, as demonstrated by the SSA simulation of therapy 30, whose results are shown in Figure 6B.

If a general unstructured multiplicative uncertainty (namely, a full Δ matrix) had been included in the model, an analysis of performance would have produced conservative results and some robust therapies might have been discarded. In fact, this definition of uncertainty is not directly connected to the physical phenomena occurring in the system and will generally include physically unfeasible perturbations. Defining ranges for parameter values includes a structured uncertainty in the model, preserving a closer physical interpretation, related to the stochastic noise and the experimental error inherent to biological networks.

**Figure 7:**
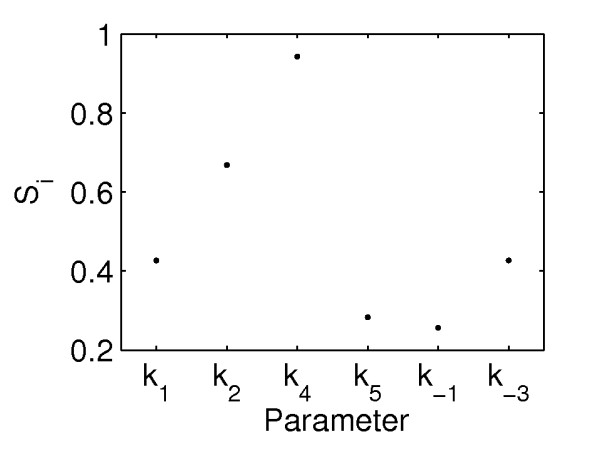
**Local sensitivity analysis results**. Sensitivity coefficients, *S_i_*, of the diseased output at steady-state, *y_ss,d_*, respect to the parameter indicated on the *x*-axis. The coefficients are normalized with the nominal value of each parameter and with the steady-state output concentration *y_ss,d_*.

Sensitivity analysis is a possible alternative approach to identify parameters for therapy design. For comparison, the local sensitivity analysis (LSA) of the case study is reported in Figure 7. Sensitivity coefficients with respect to only one parameter were calculated, even if global sensitivity analysis methods exist [40]. Parameter *k_3 _*was not included, as it is not present in the linearized version of the model used in the SSV analysis. LSA results show the disease state is particularly sensitive to parameters *k_2 _*and *k_4_*, those involved in the single target therapies 2 and 3. Therapy 2 resulted in non-robust performance (Figure 6A), and therapy 3 failed even the nominal performance test, when parametric uncertainty is not accounted for (results not shown). The discrepancy in the results is due to the local character of LSA. Even when the model is linear, LSA is unable to account for system behaviour in the presence of large perturbations. Moreover, as it does not include a definition of performance, once sensitive parameters are identified, the information on the amount of perturbation needed to restore a healthy performance is not available.

Several extensions of SSV analysis exists which can be invaluable to drug discovery. As parameter fitting can be computationally expensive, by reversing the idea of robust performance and searching for target combinations which most easily *destabilize *the diseased state, the number of parameter fittings performed early in the analysis can be greatly reduced [34]. Furthermore, in this paper we considered a single-input, single-output system with uncertainty being limited to the interactions (parameters) of the network. In multiple input and multiple output (MIMO) networks, more complex performance envelopes can be considered during robust performance analysis. The analysis can also be extended to include other clinically-interesting sources of uncertainty, such as dosages issues, blood clearance, etc.

## Conclusions

The complexity and size of biological systems make observation-based approaches to combinatorial drug therapy discovery prohibitive due to the associated financial burden and time requirements. Many companies are now aware of the value of using *in silico *techniques to guide discovery, but these analyses may rely too heavily on model accuracy. Using tools such as SSV analysis, biological networks can be screened for MDTs that are robust to various uncertainties. These uncertainties may be noise experienced in data, neglected dynamics, or even intrinsic biological variability. Furthermore, the performance of the network can be user-defined to cover several drug-related concerns such as drug efficacy and known potential side effects. MDTs identified by SSV analysis are robust to all model hypotheses expressed in the uncertainty description, and are more likely to be effective during experimentation. In conclusion, SSV analysis can prioritize target combinations by quantifying treatment efficacy given uncertainty in a systematic way.

## Methods

### Nominal model

All the numerical simulations were performed in MATLAB 7.7.0 R2008b (MathWorks, Inc.). The healthy and diseased steady-states were calculated using Matlab function *fsolve *to solve the system of equations in Figure 2B after equating to zero the time derivative terms. The nonlinear ODE model in Figure 2B was solved using Matlab function *ode15s*.

### Stochastic Simulation Algorithm (SSA) and performance envelopes

The nonlinear model was expressed in terms of number of molecules, instead of concentrations, and SSA was applied according to [37]. The initial conditions for the results shown in Figure 3A were the healthy and diseased steady-states, respectively. 100 trajectories were generated and interpolated at 100 regular time points. At each time point mean and standard deviation values among the trajectories were calculated.

The upper, *y_ub_*, and lower, *y_lb_*, bounds of the healthy performance envelope shown in Figure 3A were then calculated by:

(7) $$\left\{ \begin{array}{l} y_{lb}=y_{ss,h}-f\cdot s_{mean}\\ y_{ub}=y_{ss,h}+f\cdot s_{mean} \end{array}\right.,$$

where *y_ss,h _*is the healthy output at steady-state, *s_mean _*is the time-averaged standard deviation from the stochastic simulations, and *f *is a weighting factor chosen to have the stochastic envelopes reasonably contained in the performance ones. A value of *f *equal to 1.6 was used. The diseased performance envelope shown in Figure 3A was calculated analogously.

### Derivation of potential therapies

Each therapy is obtained by fitting the output of the nonlinear model with diseased input, *u_tot,d_*, to the healthy output response. The fitting was performed using Matlab function *fmincon *by minimizing the following cost function, *C*:

(8) $$C=\sum_{i=1}^{20}{\left[y_{dt}(t_{i})-y_{h}(t_{i})\right]}^{2},$$

where *t_i _*are 20 regularly-spaced time points in the simulated time span. The initial conditions were given by the diseased steady-state. Up to 4 parameters were simultaneously allowed to change in the range ±100% of their nominal value.

### Test for nominal performance

The model of each therapy was run using the healthy steady-state as initial condition. The absolute deviation of the therapy model output from the healthy one was calculated, and the therapies that met the condition in (2) at each time point, *t_i_*, defined above, were selected as respondent to the requirements of nominal performance.

### Model linearization

The model of each therapy was transformed by analytical Jacobian linearization around the healthy steady-state. The following deviation variables, indicated by the over bar, were defined: $\bar{x}=x-x_{ss,h}$, $\bar{ux}=ux-ux_{ss,h}$, $\bar{y}=y-y_{ss,h}$, $\bar{u_{tot}}=u_{tot}-u_{tot,h}$. Then, the model was rearranged in state-space form:

(9) $$\left\{ \begin{array}{l} \frac{dz}{dt}=Az+Bv\\ w=Cz+Dv \end{array}\right.,$$

where $z={\left[\bar{x},\bar{ux},\bar{y}\right]}^{T}$, $v=\bar{u_{tot}}$, $w=\bar{y}$, and *A*, *B*, *C*, and *D *are constant matrices. As *A*, *B*, *C*, and *D *depend on the parameter values, they are different for each therapeutically-treated diseased model and for the healthy one. Furthermore, the therapy models have input $\bar{u_{tot,d}}=u_{tot,d}-u_{tot,h}$, while the healthy one has null input in deviation variables.

### Model rearrangement and SSV analysis

An uncertainty *r_k _*= 45% was applied to each parameter in the model, according to the definition in (3). Multiple deviation models were then defined as the difference between each therapy model and the healthy one in the form described by (9). In practice these deviation models were obtained numerically in Matlab. Their output was normalized by the performance weighting factor, *w_p_*, defined in (3). The models were numerically converted into an M-Δ form by using the *Robust Control Toolbox *in Matlab.

SSV analysis was applied to all the deviation models, after a nominal stability check (data not shown). *μ_RP _*was calculated using Matlab function *mussv*.

## Authors' contributions

CL participated in the design of the study, carried out the numerical simulations and drafted the manuscript. JES participated in the design of the study and helped to draft the manuscript. KRS helped with preliminary stochastic analyses in the design of the manuscript. LRP participated in the design of the study and helped to draft the manuscript. FJD participated in the design of the study and helped to draft the manuscript. All authors read and approved the final manuscript.
